# Linking Intrinsic Filler Properties to Gas Separation Performance in Polyimide-Based Mixed-Matrix Membranes

**DOI:** 10.3390/polym18131645

**Published:** 2026-07-01

**Authors:** Alba Torres, Cenit Soto, Javier Carmona, Raúl Muñoz, Laura Palacio, Pedro Prádanos, Alberto Tena, Antonio Hernández

**Affiliations:** 1 Surface and Porous Materials (SMAP), Associated Research Unit to CSIC, Facultad de Ciencias, Universidad de Valladolid, Paseo Belén 7, 47011 Valladolid, Spain; alba.torres@uva.es (A.T.); marveliacenit.soto@uva.es (C.S.); fcojavier.carmona@uva.es (J.C.); laura.palacio@uva.es (L.P.); ppradanos@uva.es (P.P.); a.tena@uva.es (A.T.); 2 Institute of Sustainable Processes (ISP), Universidad de Valladolid, Paseo Prado de la Magdalena 3-5, 47011 Valladolid, Spain; raul.munoz.torre@uva.es

**Keywords:** mixed-matrix membranes, porous organic polymers, gas separation, free volume, d-spacing

## Abstract

Mixed-matrix membranes (MMMs) incorporating porous organic fillers into high-performance polyimides were developed to investigate the influence of free volume and molecular architecture on gas transport. Four structurally rigid, intrinsically porous fillers (TFAP-Trp, Is-Trp, TFAP-TPB, and Is-TPB) were incorporated into a range of polymer matrices (P84^®^, Matrimid^®^, Pi-DAPOH, Pi-DAROH, Pi-HABAc, Pi-DAM, and PIM-1), enabling the development of a matrix-independent methodology for estimating intrinsic filler permeabilities for five gases (He, O_2_, N_2_, CH_4_, and CO_2_). This comprehensive multi-matrix, multi-gas study reveals a strong correlation between filler fractional free volume (FFV), BET surface area, and gas permeability, with isatin-based fillers exhibiting particularly high CO_2_ permeability. Filler incorporation generally resulted in substantial permeability enhancements (100–350%) while maintaining selectivity, often with only minor losses or even favorable improvements in CO_2_/CH_4_ and He/CH_4_ separation performance. Several MMMs, particularly those based on Pi-DAPOH and Pi-DAROH polyimides, approached or exceeded the Robeson upper bound. Analysis of permeability as a function of gas kinetic diameter further elucidated clear structure–property relationships, confirming that filler-induced disruption of polymer chain packing and the creation of additional transport pathways are the primary factors governing separation performance. Overall, these findings demonstrate that rationally designed porous organic fillers provide a robust and broadly applicable strategy for mitigating the permeability–selectivity trade-off in polymer membranes and enhancing gas separation efficiency.

## 1. Introduction

The development of high-performance membranes for gas separation is being increasingly centered on mixed-matrix membranes (MMMs), which integrate a continuous polymer matrix with a dispersed porous filler [1]. Despite their considerable potential, one of the principal challenges in MMM fabrication remains the achievement of strong interfacial compatibility between the polymer and the filler. Insufficient interfacial adhesion may result in the formation of non-selective voids or rigidified interphases, both of which can adversely affect gas transport properties and mechanical stability [2]. Consequently, the nature of polymer–filler interactions largely determines whether the resulting hybrid material exhibits synergistic performance enhancements or suffers from transport limitations [3].

Although significant advances have been made in MMM development, systematic investigations involving multiple polymer matrices, each combined with a common family of fillers, remain scarce. Most studies have focused on optimizing either the polymer matrix [4,5] or the filler phase [6,7,8] independently, making it difficult to disentangle the specific contributions of polymer–filler interactions to membrane performance. Furthermore, many reports focus on commercial polymers such as Matrimid^®^ [9,10,11], whose gas transport properties are characterized by moderate selectivities and relatively low permeabilities. A comprehensive understanding of interfacial phenomena therefore requires comparative studies in which the same filler is incorporated into different polymer matrices. Conversely, different fillers are evaluated within the same matrix. Such an approach enables the identification of the key structural and physicochemical parameters governing gas transport in MMMs.

Among the wide range of polymeric materials available for membrane fabrication, polyimides are among the most extensively studied and industrially relevant candidates for gas separation applications [12]. Their exceptional thermal and chemical stability, superior mechanical properties, and tunable free volumes make them particularly attractive for operations under demanding industrial conditions [5]. Moreover, the structural versatility afforded by the selection of dianhydride and diamine monomers enables precise control over gas transport characteristics. As a result, polyimide-based membranes have been widely investigated for applications including biogas upgrading [13,14], natural gas sweetening [15,16], and helium recovery [17].

These separations are of considerable industrial and environmental importance. Helium is a scarce and non-renewable resource whose unique properties, including low boiling temperature, high thermal conductivity, and chemical inertness, make it indispensable in cryogenic technologies, semiconductor manufacturing, medical imaging, and aerospace applications. Helium is commonly recovered from natural gas reservoirs, where it is typically present alongside methane and other hydrocarbons. Methane, the principal constituent of natural gas, frequently contains carbon dioxide impurities that reduce fuel quality, promote corrosion of processing equipment, and increase separation costs. Furthermore, carbon dioxide emissions contribute significantly to anthropogenic climate change. Similarly, raw biogas streams contain substantial amounts of carbon dioxide that must be removed to produce high-purity biomethane suitable for energy applications. Consequently, membrane materials capable of simultaneously achieving high permeability and high CO_2_/CH_4_ or He/CH_4_ selectivity are highly desirable for energy-efficient gas purification processes.

The compatibility between the polymer matrix and the filler is strongly influenced by the nature of the dispersed phase. Inorganic fillers, such as zeolites, metal–organic frameworks, and silica nanoparticles, possess well-defined porosity and high intrinsic permeability that can significantly enhance gas transport [1]. However, their rigid surfaces and often hydrophilic character frequently result in poor dispersion and interfacial defects when incorporated into predominantly hydrophobic polymer matrices. Surface functionalization and coupling agents are commonly employed to improve compatibility, but these approaches increase synthetic complexity and may partially compromise the intrinsic porosity of the filler [18].

Organic fillers, by contrast, generally exhibit improved compatibility with polymeric matrices owing to their similar chemical composition and greater mechanical compliance [19]. Enhanced interfacial adhesion and dispersion reduce the likelihood of defect formation and non-selective transport pathways. Nevertheless, many organic fillers lack the permanent porosity and structural rigidity necessary to significantly improve molecular sieving performance.

Porous organic polymers (POPs), also referred to as porous polymer networks (PPNs), represent a promising class of materials capable of bridging the advantages of inorganic and organic fillers [20]. POPs combine high surface areas and tunable pore architectures with an entirely organic framework, thereby offering excellent compatibility with polymer matrices [21,22,23]. Their modular chemical structure enables the rational tailoring of polymer–filler and gas–filler interactions, facilitating the design of MMMs with optimized interfacial morphology and enhanced separation performance. Consequently, POP-based MMMs constitute an attractive strategy for overcoming longstanding compatibility challenges while exploiting the intrinsic porosity necessary for efficient gas transport. The integration of POPs within polyimide matrices therefore offers a viable route toward membranes capable of simultaneously achieving high permeability and selectivity for applications such as biogas upgrading, natural gas purification, and helium recovery.

In the present work, four porous organic polymers were synthesized and incorporated into seven polymer matrices, including the commercial polyimides P84^®^ and Matrimid^®^; the laboratory-synthesized polyimides Pi-HABAc, Pi-DAPOH, Pi-DAROH, and Pi-DAM; and the high-free-volume polymer PIM-1. The resulting MMMs were systematically characterized in terms of their physicochemical properties and gas separation performance, providing a comprehensive assessment of polymer–filler interactions across a broad range of membrane architectures.

## 2. Materials and Methods

P84^®^ was purchased from HP Polymer GmbH (Lenzing, Austria). Matrimid^®^ was purchased from Huntsman Advanced Materials GmbH (Berkamen, Germany). The other polymeric matrices were synthesized by us following the synthetic protocols that can be found in our previous work [24].

The fillers TFAP-Trp and Is-Trp were obtained by the reaction of triptycene (Trp; Fluorochem, Glossop, UK) with 2,2,2-trifluoroacetophenone (TFAP; Apollo Scientific, Manchester, UK) or isatin (Is; Alpha Aesar, Ward Hill, MA, USA), respectively, in the presence of trifluoromethanesulfonic acid (TFSA; Apollo Scientific, Manchester, UK). The fillers TFAP-TPB and Is-TPB were obtained in the same conditions by the reaction of 1,3,5-triphenylbenzene (TPB; Alfa Aesar, Ward Hill, MA, USA) with TFAP or Is respectively. The corresponding methodology was established elsewhere [25].

Number-average (Mn) and weight-average (Mw) molecular weights were determined by gel permeation chromatography (GPC). Polyimides were analyzed in DMF containing 0.1 wt.% LiBr using a Waters chromatograph equipped with a 2414 refractive index detector and Styragel HR3 and HR5 columns (Waters, Milford, MA, USA). Calibration was performed with polystyrene standards. PIM-1 was analyzed in THF using HR4, HR1, and HR0.5 Waters columns. The corresponding Mn and Mw values are shown in Table S1.

A summary of the synthetic protocol as well as the carbon solid-state magnetic resonance spectra (^13^C_solid_-NMR) and FTIR are shown in the Supplementary Materials (Figures S1–S3). It is worth noting that no significant differences were observed in the intensity of the peaks, nor were any new signals detected. This must have been due to the low particle content that is clearly insufficient to see any of the signals attributed to the POP or filler–particle interaction.

### 2.1. Mixed-Matrix Membrane Formation

Pristine membranes and MMMs were prepared by solvent casting method. For pristine membranes, 500 mg of polymer was dissolved in the appropriate solvent and filtered through a 0.45 μm syringe filter. THF was used for Matrimid^®^ and PIM-1, while NMP was employed for the remaining polymers.

For MMM fabrication, the elimination of particle aggregates and the achievement of homogeneous particle dispersion are crucial for obtaining high-performance membranes. Previous studies that employed a 10% POP content reported good filler–particle compatibility with no evidence of interfacial voids or particle agglomeration.

Accordingly, for MMMs containing 10 wt.% filler, the polymer solution and POP suspension were prepared separately. After polymer dissolution and filtration, the POP suspension was sonicated for 20 min (40 cycles of 20 s at 30% amplitude separated by 10 s pauses) using a S250D digital sonifier (Branson Ultrasonics, Brookfield, CT, USA) to ensure homogeneous dispersion. The polymer solution and filler suspension were then combined and stirred for 4 h before being cast onto leveled glass plates using 9 cm diameter glass rings.

THF-cast membranes were dried at room temperature for 12 h followed by 12 h under vacuum at 120 °C. NMP-cast membranes were dried sequentially at 60 °C (12 h), 80 °C (8 h), and 100 °C (1 h), followed by vacuum drying at 200 °C for 8 h and thermal treatment up to 300 °C under an inert atmosphere.

Membrane thickness was measured using a Dualscope MP0R (Fischer, Waldachtal, Baden-Wurttemberg, Germany), yielding an average thickness of 45 ± 12 μm.

### 2.2. Characterization Techniques

^13^C_solid_-NMR spectra were recorded for the fillers’ structural determinations with a solid-state Bruker Avance 400 instrument (Bruker, Mannheim, Germany) working at 100.6 MHz. All membranes and POPs were submitted to Attenuated Total Reflectance–Fourier Transform Infrared (ATR-FTIR) spectrometry to confirm the presence of characteristic functional groups. All spectra were performed using a PerkinElmer Spectrum One FT-IR instrument (PerkinElmer, Waltham, MA, USA) from 4000 to 400 cm^−1^ and with a 4 cm^−1^ resolution. These techniques confirmed that the target polymers were effectively synthesized as shown in the Supplementary Materials.

Wide-angle X-ray scattering (WAXS) analysis was performed to study the preferential packing of MMMs by the determination of the so-called d-spacing, $d_{s}$, according to Bragg’s Law:

(1) $$n\lambda ={2d}_{s}\;sin(\theta )$$

where *n* is an integer, *λ* is the X-ray wavelength, and *θ* is the incidence angle. X-ray diffraction (XRD) patterns were recorded at room temperature using a D8 Discover A25 diffractometer (Bruker, Billerica, MA, USA) equipped with a Göbel mirror and Cu Kα radiation (λ = 1.542 Å). Measurements were performed over a 2θ range of 0–70° with a step size of 0.020° and a counting time of 0.5 s per step.

In polymers, the d-spacing represents the average distance between repeating structural features and provides information on chain packing, free volume, and molecular organization, all of which influence gas transport properties.

The fractional free volume, $f^{i}$, for the *i*-th filler is

(2) $$f^{i}=\frac{V^{i}-V_{0}^{i}}{V^{i}}.$$

where $V^{i}\;$is the total specific volume, while $V_{0}^{i}$ is the occupied volume of the polymer. This occupied volume can be taken as the zero-point (i.e., at 0 K) specific volume, which is closely related to the van der Waals volume as

(3) $$V_{0}^{i}=1.3V_{W}$$

where $V_{W}$ is the van der Waals volume that can be calculated by molecular modeling using the Materials Studio software (DS BIOVIA Materials Studio 2023 v23.1.0.3829).

The fillers’ specific volumes were evaluated as the sum of their skeletal specific volumes, $V_{sk}^{i}$, plus the specific volumes within the pores of the fillers, $V_{p}^{i}$:

(4) $$V^{i}=V_{sk}^{i}+V_{p}^{i}$$

The skeletal volume, $V_{sk}^{i}$, was determined by gas displacement using the volume–pressure relationship of Boyle’s law, with helium used as the displacement medium. The sample was placed in a sealed cup of a known volume (2.5 cm^3^). Gas was introduced to the sample chamber and then expanded into a second empty chamber with a known volume. The pressure observed after filling the sample cell and the pressure discharged into the expansion chamber were measured, and then the volume was calculated. An AccuPyc 1330 V 2.04 N (Micromeritics Instrument Corporation, Norcross, GA, USA) pycnometer was used. The specific volume was determined by dividing the sample weight by the volume measured.

The specific volume within the pores of the fillers was measured by CO_2_ adsorption at 0 °C (273 K) in a volumetric device, the Autosorb iQ (Quantachrome Instruments, Boynton Beach, FL, USA). Samples were degassed at 125 °C for 18 h under vacuum before the CO_2_ adsorption measurements. The adsorption isotherm data were used to obtain the pore size distribution by the nonlocal density functional theory equilibrium model (NLDFT). Acquisitions and calculations were carried out using the Quantachrome^®^ ASiQwin software (version 5.21). Finally, Equations (2)–(4) allowed the evaluation of the fraction of free volume (FFV).

Thermogravimetric Analysis (TGA) was carried out with a TA Instruments-Water Corp. TGA 550 thermogravimetric analyzer (TA Instruments, Milford, MA, USA) with a ramp of 10 °C/min from 30 to 800 °C and 40 mL/min N2 flux. Differential Scanning Calorimetry (DSC) was carried out in a TA Instruments modulated DSC-25 analyzer (TA Instruments, Milford, MA, USA) to determine the glass transition temperatures (T_g_) of the polymer matrix and the possible change in MMMs. A double heating procedure was applied: a first heating rate from 20 °C/min to 250 °C and a second heating rate of 20 °C/min up to 400 °C under a N_2_ atmosphere. TGA and DSC computational data were treated with TRIOS Software (TA Instruments v5.1.1.46572, Milford, MA, USA). The corresponding results are shown in Tables S2 and S3 of the Supplementary Materials.

ESEM images were taken using FEI Quanta 200 FEG (FEI Company, Hillsboro, OR, USA) operating at an acceleration voltage of 20 kV on Au-metallized samples.

Finally, the BET areas were measured from N_2_ adsorption isotherms measured at −196 °C (77 K) in a volumetric device, the Autosorb iQ (Quantachrome Instruments, Boynton Beach, FL, USA), in the 0.05-to-0.35 relative pressure (p/p_0_) range. The samples were degassed at 120 °C for 10 h under vacuum, before the initiation of the sorption measurements, to eliminate possible adsorbed gases or water vapor.

Single gas permeability coefficient (P_i_) measurements of He, N_2_, O_2_, CH_4_ and CO_2_ were measured at 35 °C and an upstream pressure of 3 bar for pristine membranes and MMMs using a barometric apparatus. Helium permeability was measured at three different pressures (1, 2 and 3 bar) to ensure the absence of pinholes. The permeability coefficient is typically expressed in Barrer [1 Barrer = 10^−10^ (cm^3^ (STP) cm)/(cm^2^ s cmHg) = 7.5005 × 10^−18^ m^2^ s^2^ Pa^−1^]. It was obtained according to the following equation:

(5) $$P=\frac{273}{76}\left(\frac{LV}{Tp_{a}A}\right)\frac{dp}{dt}.$$

where L is the thickness of the membranes, V is the downstream volume, T is the temperature, $p_{a}\;$is the pressure of the feed gas, A is the effective area, dp/dt is the slope of downstream versus time. The factors refer to standard pressure and temperature (76 cm Hg and 273.15 K). The ideal selectivity for a determined gas pair was calculated as the ratio of their single gas permeabilities. The measurements have an associated error below 5%.

It has been proved [19,24] that there is a common tendency for $\log\left(P_{MMM}/P_{matrix}\right)$ to decrease linearly versus $\log\left(P_{matrix}\right)\;$for all the gases for low filler contents. Consequently:

(6) $$\log\frac{P_{MMM}}{P_{matrix}}=\log C_{1}-C_{2}\log\left(P_{matrix}\right)$$

with $C_{2}>0$.

In accordance with Equation (6),

(7) $$\frac{P_{MMM}}{P_{matrix}}=C_{1}{\left(P_{matrix}\right)}^{-C_{2}}$$

or

(8) $$P_{MMM}=\frac{C_{1}}{{\left(P_{matrix}\right)}^{C_{2}-1}}$$

Note that Equation (8) means that the addition of small amounts of a highly permeable filler is specifically beneficial for the low permeability polymeric matrices (assuming $C_{2}>1$). If, on the contrary, $C_{2}\ll 1$, then

(9) $$P_{MMM}≃C_{1}P_{matrix}$$

The permeability of dual-phase systems, when the content of the disperse phase is low enough, can be adjusted or predicted by some simple models [26]. Certain models are exclusively intended to account for low filler contents within the continuous matrix. For example, the Bruggeman, Bötcher, and De Loor models give different functions of the volume fraction of the filler, ϕ [27]:

(10) $$P_{MMM}=P_{matrix}{\left(1-\phi \right)}^{-3}$$

(11) $$P_{MMM}=P_{matrix}{\left(1-\phi \right)}^{-1}$$

(12) $$P_{MMM}=P_{matrix}\left(1-\phi \right){\left(1-2\phi \right)}^{-1}$$

The corresponding multiplicative constants would be 1.37, 1.11 and 1.13, respectively, for ϕ=0.10. If Equations (9)–(12) are compared, the slope of $\log\left(P_{matrix}\right)$ versus $\log\left(P_{MMM}/P_{matrix}\right)$ should be $0.05<\log C_{1}<0.14$. These slopes would depend on the content of the filler in the MMM; for example, for 30%, Equations (10)–(12) would give $0.15<\log C_{1}<0.47$ if such linear dependence could still be assumed.

Note, as well, that the straight lines predicted by Equation (6) can be extrapolated for high permeabilities to cross the line defined by $P_{MMM}=P_{matrix}$ ($\log\left(P_{MMM}/P_{matrix}\right)=0$). This intercept should correspond to the permeability of the pure microporous organic polymer.

Conversely, the relationship between diffusivity (and permeability) with kinetic diameter and free volume has been widely investigated in the literature. A comprehensive review of this subject was provided by Matteucci et al. [28]. Thornton et al. [29] showed that for membranes in which solubility is nearly independent of the fraction of free volume, that is, where transport is governed primarily by diffusion:

(13) $$P=SD=Ae^{Bf}$$

Meanwhile, various forms of the dependence of the constant, B, on the kinetic diameter, $d_{k}$, have been proposed. However, it has been demonstrated that a quadratic dependence is generally sufficient [30,31]:

(14) $$B=a+bd_{k}+cd_{k}^{2}$$

Combining Equations (13) and (14), we obtain

(15) $$\ln P=\left[\ln A+af\right]+\left[bf\right]d_{k}+\left[cf\right]d_{k}^{2}$$

## 3. Results

### 3.1. Free Volume of the Fillers

**Figure 1:**
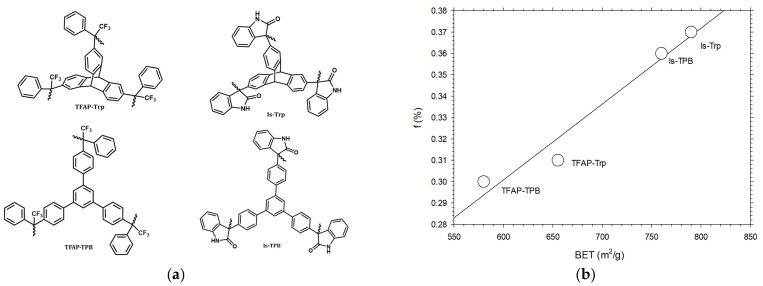
(**a**) Porous organic polymers‘ (POPs) chemical structure. (**b**) Fractional free volume (FFV) as a function of the BET area for the fillers used in this work.

The chemical structures of the porous fillers are shown in Figure 1a, while Figure 1b presents their fractional free volumes and BET surface areas. A nearly linear correlation can be observed between these two parameters, indicating that increased free volume is associated with higher surface area. Although this trend is expected, the relationship is also influenced by pore geometry, as similar void volumes could result in substantially different surface areas accessible to adsorption.

**Figure 2:**
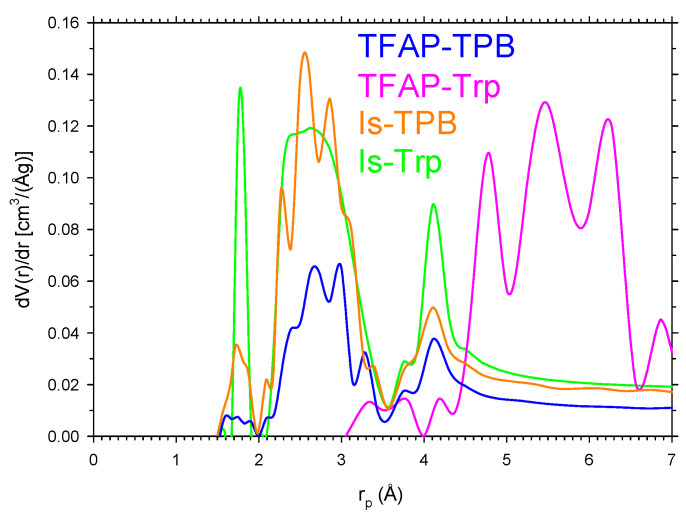
Pore radius distributions of the fillers as obtained from CO_2_ adsorption.

Some pore size distributions obtained from CO_2_ adsorption isotherm data by NLDFT are shown in Figure 2. The micropores presented by the fillers are always quite similar in the 2 to 3.5 Å range for the fillers containing TPB. The TFAP-Trp POP contains a significant population of bigger pores (up to 8 Å), while Is-Trp presents a big population of smaller pores (around 1.8 Å). But it is worth noting that these very large and very small pore populations are likely outside the size range that significantly contributes to gas transport.

### 3.2. Permeability and d-Spacing

**Figure 3:**
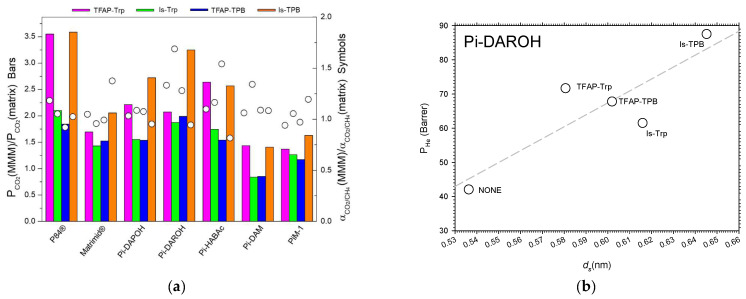
(**a**) Relative variation in the CO_2_ permeability (bars) and CO_2_/CH_4_ selectivity (symbols) of mixed-matrix membranes (MMMs) relative to the neat matrix for TFAP-Trp (purple), Is-Trp (green), TFAP-TPB (blue) and Is-TPB (orange). (**b**) Helium permeability versus d-spacing for Pi-DAROH pristine polymer and MMMs (the line serves only as a visual guide).

The permeability coefficients obtained from gas transport measurements for both the neat membrane and all MMMs are summarized in Table S4. Figure 3a summarizes the impact of incorporating the porous fillers developed in this work on the gas transport properties of the resulting MMMs. This figure reports the relative changes in effective CO_2_ permeability and CO_2_/CH_4_ selectivity with respect to the corresponding pristine polymers. Across all systems, the introduction of the fillers leads to a marked enhancement in CO_2_ permeability, while CO_2_/CH_4_ selectivity remains generally unchanged or even exhibits modest improvement.

Among the materials investigated, MMMs containing Is-TPB consistently exhibit the most pronounced permeability enhancement. This trend is fully consistent with the higher free volume and BET surface area of Is-TPB, among other factors, as shown in Figure 1b. The effect is particularly notable for PIM-1, the most permeable polymer matrix in this study, which exhibits high intrinsic CO_2_ permeability. Incorporation of Is-TPB increases the CO_2_ permeability of PIM-1 to values exceeding 9600 Barrer. For less permeable polymers, the relative improvements are even more significant. At a loading of 10 wt.% Is-TPB, the permeability enhancement follows the order: P84^®^ (358%) > Pi-DAROH (325%) > Pi-DAPOH (275%) > Pi-HABAc (257%) > Matrimid^®^ (205%) > PIM-1 (163%) > Pi-DAM (140%). Even in the least responsive system, Pi-DAM, the increase in permeability remains substantial (>140%). Despite these large changes in permeability, the CO_2_/CH_4_ selectivity of the Is-TPB MMMs exhibits minimal deviation from that of the pristine polymers, except for Matrimid^®^–Is-TPB, where a significant selectivity improvement can be observed.

The TFAP-Trp filler also yields notable enhancements in CO_2_ permeability for several matrices, including P84^®^, Pi-HABAc, and Pi-DAM, in some cases reaching levels comparable to those obtained with Is-TPB. Although TFAP-Trp presents only moderate free volume and surface area values, it provides the second largest improvement in overall gas separation performance. This beneficial effect may be attributed to both the presence of –CF_3_ groups and the substantial disruption of polymer packing induced by the triptycene unit [11,32,33,34]. This can be rationalized by comparing TFAP-Trp with TFAP-TPB: both fillers incorporate –CF_3_ groups and exhibit similar free volume characteristics (Figure 1b). However, the triptycene core produces a stronger packing distortion than the triphenylbenzene structure.

For the remaining MMMs, permeability enhancements generally fall within the range of 100–200% relative to pristine polymers. Only in the case of Pi-DAM combined with Is-Trp or TFAP-TPB are the improvements more modest, around 75%. This low effect could be attributed to interfacial defects or non-ideal compatibility between matrix and filler. Although, in fact, Is-Trp and TFAP-TPB systematically produce the lowest permeability enhancements across nearly all polymer matrices investigated, indicating that the observed behavior is more strongly related to the intrinsic characteristics of these fillers than to a specific matrix–filler incompatibility. This trend is particularly evident for P84^®^, which exhibits the lowest intrinsic permeability and the largest differences between fillers.

For Pi-DAM and PIM-1 polymers, the relative permeability enhancement is lower than that observed for the other polyimides because the pristine polymers already possess a comparatively high permeability and free volume content. Consequently, the additional transport contribution introduced by the porous filler represents a smaller relative increase than in slower and more densely packed matrices such as P84^®^ or Matrimid^®^.

Overall, these results clearly demonstrate the effectiveness of incorporating rigid, porous organic fillers into polymer matrices to substantially boost CO_2_-transport performance without compromising selectivity.

Figure 3b presents a detailed comparison of helium permeability as a function of intersegmental distance for pristine Pi-DAROH and the corresponding MMMs containing TFAP-Trp, Is-Trp, TFAP-TPB, and Is-TPB fillers.

**Figure 4:**
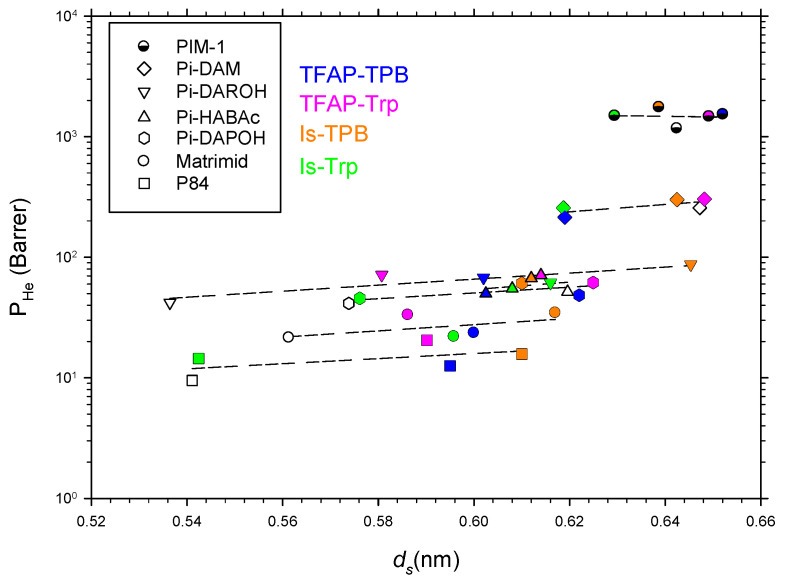
Helium permeability as a function of d-spacing for the different fillers.

It seems clear that there is a steep increase in helium permeability when d-spacing increases, confirming the positive impact of the inclusion of the porous fillers in the polymeric matrix. Similar behavior was observed for all the other gases tested. The resulting d-spacings were obtained from the corresponding amorphous halos shown in Figure S4. In general, permeability and $d_{s}$, as shown in Figure 4, increased when porous fillers were added to the pristine polymers with relatively low permeability. On the other hand, increasing d-spacing had a limited effect on permeability for the high-permeability polymeric matrices. In such cases, the polymer already possesses a highly open microporous structure, limiting the ability of the porous fillers to further increase the free volume. Consequently, filler-induced interfacial voids and packing disruption have a less pronounced effect on permeability than in more densely packed polymer matrices.

An in-depth study of the changes in permeability can be carried out by analyzing the diffusion and solubility coefficients according to Equation (13). The diffusion coefficient (D) can be determined from time-lag permeation measurements as D=L26θ, where L is the membrane thickness and θ is the time lag obtained by extrapolating the linear region of the permeation curve to the x-axis. Once P and D are evaluated, solubility (S) can be determined using Equation (13). The evaluated values of D and S with the different filler particles are reported in Figure S5. Regarding solubility coefficients, no significant variations can be observed, Is-TPB being the particle that exhibits the highest solubility improvement, which could be related to its high BET area. On the other hand, a noticeable increase in diffusivity for every particle inclusion can be observed except for Matrimid^®^. Comparing Trp-based particles, the one containing TFAP offers a higher enhancement in diffusivity than the one containing Is. This could be because the –CF_3_ groups in TFAP may prevent efficient chain packing, creating wider or additional transport pathways. For the TPB-based fillers, a reverse tendency is revealed, where Is-TPB offers higher diffusivity than TFAP-TPB. In this situation, possible better packing of the functional groups between TFAP and TPB could explain fewer diffusion channels which align with the low BET area. Thus, it is the TFAP-TPB particle which exhibits the least improvement in agreement with the low permeability results.

**Figure 5:**
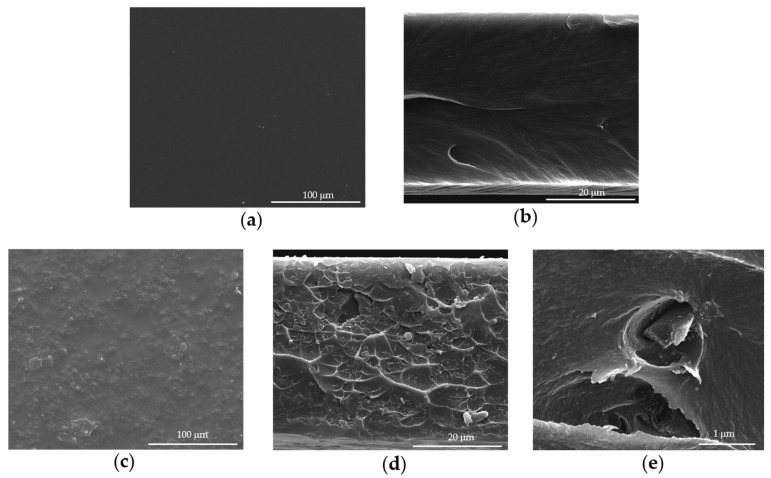
Surface and cross-fraction images of Pi-DAPOH (**a**,**b**) and Pi-DAPOH–Is-TPB (**c**–**e**).

ESEM images were obtained to analyze the compatibility and homogeneity of the MMMs obtained. Figure 5 shows the surface and cross-section of Pi-DAPOH and Pi-DAPOH–Is-TPB. For the neat Pi-DAPOH membrane, a dense, homogeneous, defect-free cross-section can be observed. For the MMM, surface images show no macroscopic defects and reveal dispersed particles which are coated by the polymer matrix. In the cross-section, the Is-TPB morphology appears to be composed of rough amorphous grains. A good compatibility between Is-TPB and Pi-DAPOH can be observed, as the grains appear to be embedded and fully coated by the polymer. In Figure 5e, an 800 nm particle can be observed with a narrow interfacial gap. In addition, Is-TPB particles are homogeneously dispersed throughout the membrane thickness. This distribution is consistent with the higher permeability increase for Is-TPB–MMMs, where a controlled increase in free volume and modified chain packing is induced without compromising selectivity.

**Figure 6:**
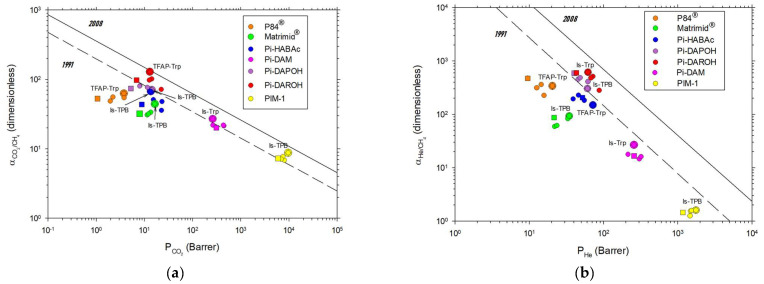
Separation performances for various gas pairs: (**a**) CO_2_/CH_4_ selectivity vs. CO_2_ permeability; (**b**) He/CH_4_ selectivity vs. He permeability. The pristine polymer matrix membranes correspond to the squares, and the best MMM results for each polymeric matrix are labelled.

A compact evaluation of the effective improvement in separation performances can generally be observed by the so-called Robeson upper bounds by representing the gas pair selectivity vs. permeability of the faster gas in a logarithmic scale [35,36]. In this way, Figure 6a represents the selectivity for the CO_2_/CH_4_ pair versus the CO_2_ permeability for the membranes used. Square symbols correspond to the pristine polymer matrix membranes. Moreover, the best MMM results (best selectivity vs. permeability compromise) for each polymeric matrix are labelled. In Figure 6b, the corresponding results for the He/CH_4_ pair are shown.

As discussed above, the incorporation of porous organic network fillers generally enhances membrane permeability, largely irrespective of filler type. This trend can be observed even for highly permeable matrices such as PIM-1, highlighting the robustness of this approach. The addition of microporous fillers promotes gas diffusion, resulting in an overall increase in permeability [37].

A notable exception can be observed for the Is-TPB filler, whose incorporation frequently leads to simultaneous improvements in both permeability and selectivity. By contrast, the filler exhibiting the highest intrinsic porosity, Is-Trp, delivers the best overall performance only when combined with Pi-DAM. In this case, the resulting Pi-DAM/Is-Trp MMM exhibits enhanced CO_2_/CH_4_ and He/CH_4_ selectivities while maintaining the permeability of the pristine polymer.

Particularly significant improvements can be observed for the hydroxyl-containing polyimides Pi-DAPOH and Pi-DAROH, which contain one and two hydroxyl groups per repeating unit, respectively. These polymers are known to exhibit intermolecular hydrogen bonding [38,39]. The incorporation of porous fillers is expected to disrupt chain packing and partially weaken these interactions, generating additional free volume and facilitating gas transport. In any case, Pi-DAROH and Pi-DAPOH give the best results, especially Pi-DAROH, which even reaches the 2008 Robeson upper limit for the CO_2_/CH_4_ pair.

Owing to the relatively low filler loading and the thickness of the membranes, permeability enhancements are achieved while largely preserving selectivity. It should be noted, however, that this behavior may differ in ultrathin selective layers, where interfacial effects become more pronounced. Indeed, the fabrication of defect-free thin-film MMMs remains one of the major challenges in membrane science.

### 3.3. Determination of the Porous Filler Gas Separation Performances

**Figure 7:**
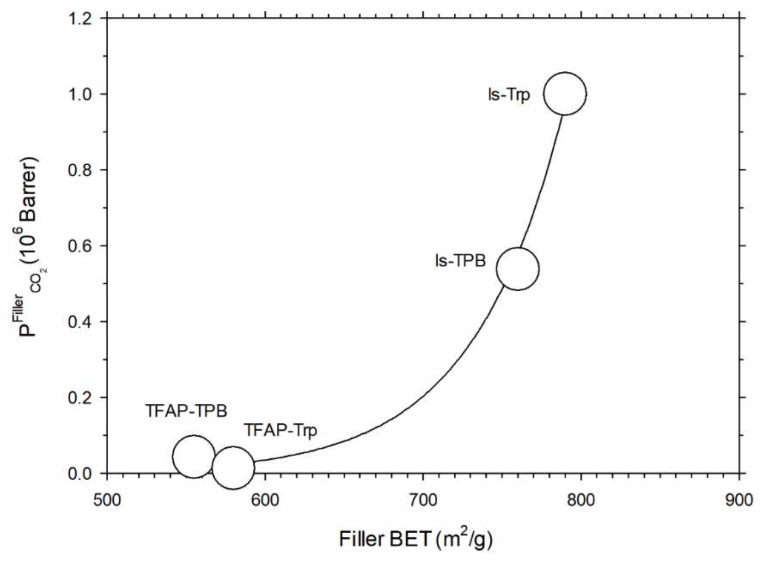
Extrapolated CO_2_ permeability of the fillers as a function of their BET area.

The chemical reaction for the synthesis of the porous filler leads, generally, to highly stable insoluble crosslinked materials. Therefore, the processability of the material as a continuous layer on its own is limited. On the other hand, determining the gas separation performances of the fillers might help to forecast the properties of future MMMs before manufacturing them. The large amount of information provided in this work can be used for such purposes. In this way, fitting of Equation (6) can provide a fair idea of the filler’s permeability. Although the agreement of the equation is far from being perfect for all gases and polymers—an example is shown in Figure S6—it can be accepted as an estimation of the pure filler permeabilities. Actually, the procedure gives approximately equal intercepts for $P_{MMM}/P_{matrix}=1$ for each filler and gas studied when performed. These intercepts are shown along the y-axis in Figure 7.

It can be seen in Figure 7 that the calculated CO_2_ permeability of the filler increases sharply with its BET area (which is linear with the free volume fraction, as shown in Figure 1). This work presents an innovative estimation of the intrinsic CO_2_ permeability of the porous fillers, derived from a multi-matrix analysis that leverages the gas transport response of several MMM systems incorporating the same fillers. This approach departs from traditional methodologies—typically based on indirect assumptions or single-matrix extrapolations—by using consistent trends observed across multiple polymeric hosts. As shown in the plot, the effective estimated permeability of the filler ($P_{{\mathrm{CO}}_{2}}^{\mathrm{filler}}$) scales strongly with the filler’s BET surface area (and subsequently with the fraction of free volume), revealing a clear structure–property relationship. Fillers with relatively low BET areas, such as TFAP-TPB and TFAP-Trp (≈600–620 m^2^·g^−1^), exhibit modest intrinsic permeabilities, whereas fillers with significantly larger accessible surface areas, such as Is-TPB and Is-Trp (≈750–820 m^2^·g^−1^), reach permeability values that are nearly one order of magnitude higher. This trend underscores the direct contribution of internal free volume and accessible porosity to gas transport efficiency.

**Figure 8:**
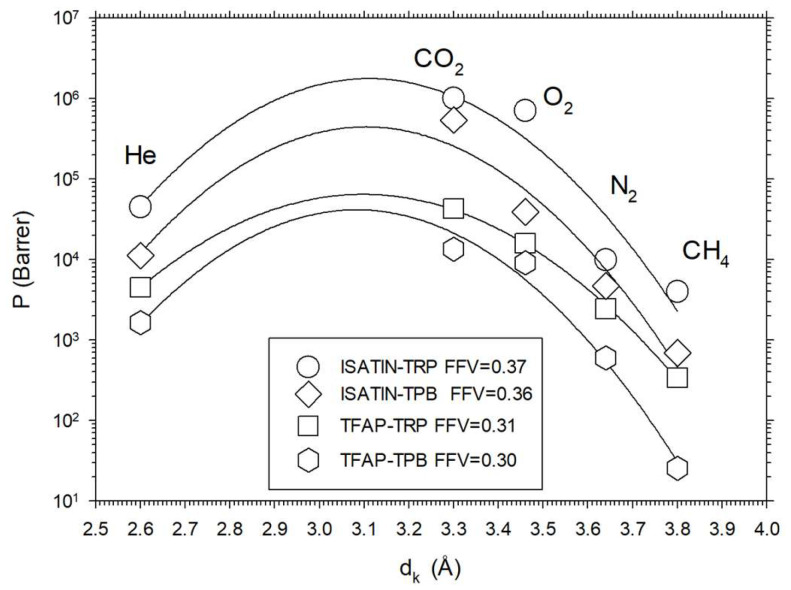
Extrapolated permeability as a function of the gas kinetic diameters for the pure POPs used here.

Similarly, the permeability coefficient for the other gases tested can be estimated. The corresponding extrapolated permeabilities (He, CO_2_, O_2_, N_2_, and CH_4_) of the fillers can be plotted against the kinetic diameter, $d_{k}$, of the gas, as shown in Figure 8.

In Figure 8, it can clearly be seen that permeability increases similarly, in the same order—TFAP-TPB, TFAP-Trp, Is-TPB, Is-Trp—of increasing free volume fraction. The shape of the fitted curves corresponds to a parabola, as predicted by Equation (15). All fillers exhibit the expected decline in permeability with increasing kinetic diameter; however, significant quantitative differences arise depending on the filler chemistry and its fractional free volume. The isatin-containing fillers, which possess the highest FFV values (0.37–0.38), consistently show the largest intrinsic permeabilities across all gases, confirming the dominant role of accessible free volume. This indicates that the transport is dominated by the diffusion mechanism. Conversely, the TFAP-containing materials, with an FFV around 0.30–0.31, present lower but still well-defined permeability profiles. The alignment of gas trends across the different fillers demonstrates that the estimation methodology captures true structure–property relationships intrinsic to the porous materials, not artefacts of individual polymers.

Beyond confirming the dominant influence of FFV on gas transport, the results also reveal a significant contribution of molecular architecture to the intrinsic permeation properties of the fillers. In particular, the triptycene-based fillers consistently exhibit higher intrinsic permeabilities than their triphenylbenzene analogues at comparable FFV values, indicating that permeability is not solely determined by free volume content but also by its spatial organization and connectivity. This behavior can be attributed to the rigid three-dimensional geometry of the triptycene motif, which promotes more efficient disruption of chain packing and facilitates the formation of interconnected diffusion pathways throughout the porous network [11].

The relative positions of the different gases further reflect variations in the underlying sorption–diffusion mechanisms governing transport. CO_2_ and O_2_ exhibit the highest permeability values as a consequence of the combined contributions of favorable diffusivity and enhanced sorption affinity, whereas the permeability of N_2_ and CH_4_ decreases more markedly with increasing kinetic diameter, reflecting stronger diffusional limitations. The simultaneous analysis of multiple gases across a range of fillers and polymer matrices not only demonstrates the robustness and general applicability of the permeability-extraction methodology but also provides a comprehensive characterization of the intrinsic transport properties of the porous fillers. Consequently, this multi-gas, multi-filler, and multi-matrix framework constitutes a significant methodological advance, enabling direct quantification and comparison of intrinsic filler permeabilities in a manner that has remained largely inaccessible in mixed-matrix membrane research.

## 4. Conclusions

The results reveal a strong correlation between FFV and BET surface area, indicating that enhanced permeability is closely associated with increased accessible free volume. Within this family of fillers, FFV and BET surface area correlate with diffusivity and solubility, respectively, supporting the use of FFV as a reliable descriptor for predicting permeability trends in mixed-matrix membranes containing highly porous fillers. Gas transport analysis further demonstrates a clear dependence of permeability on intersegmental spacing, with larger d-spacing values consistently associated with higher permeabilities for all gases studied. 

The study also demonstrates that the intrinsic CO_2_ permeability of the porous fillers is strongly governed by their porosity and accessible free volume. To quantify this relationship, a multi-matrix methodology was developed to extract intrinsic filler permeabilities from the collective transport behavior of MMMs based on different polymer matrices. By integrating information from multiple host polymers, this approach provides a more rigorous alternative to conventional estimation methods based on indirect assumptions or single-matrix extrapolations. The resulting intrinsic permeability values are consistent across diverse matrices and offer new insights into the role of filler characteristics in governing membrane transport.

Importantly, the incorporation of porous fillers generally increases permeability while maintaining, and in some cases improving, gas selectivity, thereby enhancing the overall separation performance relative to the pristine polymers. These improvements are most pronounced for fillers exhibiting high BET surface areas and FFV values, emphasizing the critical role of filler porosity in determining membrane performance. Consequently, several Pi-DAROH-based MMMs approach or exceed the Robeson upper bound for CO_2_/CH_4_ separation. The persistence of these trends across multiple polymer matrices indicates that the observed performance enhancements are primarily governed by intrinsic filler properties rather than matrix-specific effects. Overall, the combination of high free volume, favorable molecular topology, and good polymer–filler compatibility emerges as an effective strategy for mitigating the permeability–selectivity trade-off in polymer membranes.

These findings underscore the importance of controlling filler structure, accessible free volume, and polymer–filler interactions in the design of advanced mixed-matrix membranes. Further development of porous polymer networks with tailored free volume characteristics may provide new opportunities to achieve gas separation performances beyond current membrane benchmarks.

## Data Availability

All the relevant data for the research reported here are included in their entirety within the paper.

## Supplementary Materials

The following supporting information can be downloaded at https://www.mdpi.com/article/10.3390/polym18131645/s1: Figure S1: ^13^C_solid_-RMN spectra for porous fillers; Figure S2: (a) ATR-FTIR spectra for porous fillers. (b) N_2_ isotherms at 77 K of the porous fillers; Figure S3: Comparative ATR-FTIR spectra between neat polymer and the respective MMMs; Figure S4: WAXS spectra for all membranes and fillers; Figure S5: (a) Diffusion, D, and (b) solubility, S, coefficient for CO_2_ in function of particle inclusions; Figure S6: The ratio of the MMM CO_2_ permeability over the corresponding pristine membrane as a function of permeability of the pristine (matrix) membrane. The fitted straights are shown for each filler; Table S1: Number and Molecular weight and polydispersity for the synthesized polyimides, Table S2: Glass transition temperatures (T_g_) for all membranes, Table S3: Degradation temperatures (T_d_) for all membranes; Table S4: Permeability coefficients (P) along with CO_2_/CH_4_ and O_2_/N_2_ selectivity measured at 3 bar and 35 °C for all neat and MMMs. Ref. [40] is cited in the Supplementary Materials.

## Abbreviations

The following abbreviations are used in this manuscript:

MMM	Mixed-matrix membrane
FFV	Fractional free volume
POP	Porous organic polymer

## References

[1] Welton C., Chen F., Zhou H., Yi S. (2025). Carbon Capture Science & Technology Mixed Matrix Membrane Formation with Porous Metal—Organic Nanomaterials for CO_2_ Capture and Separation: A Critical Review. Carbon Capture Sci. Technol..

[2] Al-Rowaili F.N., Khaled M., Jamal A., Zahid U. (2023). Mixed Matrix Membranes for H_2_/CO_2_ Gas Separation- a Critical Review. Fuel.

[3] Goh P.S., Ismail A.F., Sanip S.M., Ng B.C., Aziz M. (2011). Recent Advances of Inorganic Fillers in Mixed Matrix Membrane for Gas Separation. Sep. Purif. Technol..

[4] Abdulhamid M.A., Ma X., Ghanem B.S., Pinnau I. (2019). Synthesis and Characterization of Organo-Soluble Polyimides Derived from Alicyclic Dianhydrides and a Dihydroxyl- Functionalized Spirobisindane Diamine. Appl. Polym. Mater..

[5] Zhang C., Cao B., Li P. (2018). Thermal Oxidative Crosslinking of Phenolphthalein-Based Cardo Polyimides with Enhanced Gas Permeability and Selectivity. J. Membr. Sci..

[6] Zong L., Li X., Cai P., Zhou H., Huang N. (2025). β-Ketoenamine Porous Organic Polymers for High- Efficiency Carbon Dioxide Adsorption and Separation. ChemSusChem.

[7] Zhang C., Liu Y., Li B., Tan B., Chen C., Xu H., Yang X. (2012). Triptycene-Based Microporous Polymers: Synthesis and Their Gas Storage Properties. ACS Macro Lett..

[8] Mondal S., Das N. (2015). Triptycene Based 1,2,3-Triazole Linked Network Polymers (TNPs): Small Gas Storage and Selective CO_2_ Capture. J. Mater. Chem. A.

[9] Tariq A.R., Tariq S.R., Sultan M., Mahmud T., Chotana G.A. (2020). Selective CO_2_ Capture through Microporous Tb(BTC)(H2O).(DMF)1.1 MOF as an Additive in Novel MMMs Fabricated from Matrimid^®^ 5218. Arab. J. Chem..

[10] González-Revuelta D., Fallanza M., Ortiz A., Gorri D. (2025). Matrimid Mixed Matrix Hollow Fiber Membranes: Influence of ZIF-8 Filler over O2/N2 Separation Performance. ACS Omega.

[11] Rizzuto C., Zhou H., Antonangelo A.R., Bezzu C.G., Carolus J., Carta M., Fuoco A. (2026). Enhancement of the Gas Separation Performance of Mixed Matrix Membranes (MMMs) with Functionalized Triptycene Hypercrosslinked Polymers of Intrinsic Microporosity (HCP-PIMs). Sep. Purif. Technol..

[12] Galizia M., Chi W.S., Smith Z.P., Merkel T.C., Baker R.W., Freeman B.D. (2017). 50th Anniversary Perspective: Polymers and Mixed Matrix Membranes for Gas and Vapor Separation: A Review and Prospective Opportunities. Macromolecules.

[13] Xu S., Zhao N., Wu L., Kang S., Zhang Z., Huo G., Dai Z., Li N. (2022). Carbon Molecular Sieve Gas Separation Membranes from Crosslinkable Bromomethylated 6FDA-DAM Polyimide. J. Membr. Sci..

[14] Alaslai N., Ghanem B., Alghunaimi F., Litwiller E., Pinnau I. (2016). Pure- and Mixed-Gas Permeation Properties of Highly Selective and Plasticization Resistant Hydroxyl-Diamine-Based 6FDA Polyimides for CO_2_/CH_4_ Separation. J. Membr. Sci..

[15] Liu Z., Liu Y., Qiu W., Koros W.J. (2020). Molecularly Engineered 6FDA-Based Polyimide Membranes for Sour Natural Gas Separation. Angew. Chem.—Int. Ed..

[16] Jusoh N., Yeong Y.F., Lau K.K., Azmi M.S. (2015). Membranes for Gas Separation Current Development and Challenges. Appl. Mech. Mater..

[17] Dai Z., Deng J., He X., Scholes C.A., Jiang X., Wang B., Guo H., Ma Y., Deng L. (2021). Helium Separation Using Membrane Technology: Recent Advances and Perspectives. Sep. Purif. Technol..

[18] Zhang Y., Nie H., Yu M., Chang Z. (2021). Post-Synthetic Modification of Tetrazine Functionalized Porous MOF for CO_2_ Sorption Performances Modulation. J. Solid State Chem..

[19] Torres A., Soto C., Carmona F.J., Sanz I., Palacio L., Prádanos P., Hernández A., Tena A. (2024). Enhancing Permeability: Unraveling the Potential of Microporous Organic Polymers in Mixed Matrix Membranes. Appl. Polym. Mater..

[20] Kuhn P., Thomas A., Antonietti M. (2009). Toward Tailorable Porous Organic Polymer Networks: A High-Temperature Dynamic Polymerization Scheme Based on Aromatic Nitriles. Macromolecules.

[21] Zhang P., Zhang C., Wang L., Dong J., Gai D., Wang W., Nguyen T.S., Yavuz C.T., Zou X., Zhu G. (2023). Basic Alkylamine Functionalized PAF-1 Hybrid Membrane with High Compatibility for Superior CO_2_ Separation from Flue Gas. Adv. Funct. Mater..

[22] Ben T., Ren H., Ma S., Cao D., Lan J., Jing X., Wang W., Xu J., Deng F., Simmons J.M. (2009). Targeted Synthesis of a Porous Aromatic Framework with High Stability and Exceptionally High Surface Area. Angew. Chemie—Int. Ed..

[23] Msayib K.J., McKeown N.B. (2016). Inexpensive Polyphenylene Network Polymers with Enhanced Microporosity. J. Mater. Chem. A.

[24] Torres A., Soto C., Carmona J., Comesaña-gandara B., de la Viuda M., Palacio L., Prádanos P., Simorte M.T., Sanz I., Muñoz R. (2024). Gas Permeability through Polyimides. Unraveling the Influence of Free Volume, Intersegmental Distance and Glass Transition Temperature. Polymers.

[25] Lopez -Iglesias B., Suárez-García F., Aguilar-Lugo C., González Ortega A., Bartolomé C., Martínez-Ilarduya J.M., De la Campa J.G., Lozano Á.E., Álvarez C. (2018). Microporous Polymer Networks for Carbon Capture Applications. ACS Appl. Mater. Interfaces.

[26] Tena A., De La Viuda M., Palacio L., Prádanos P., Marcos-Fernández Á., Lozano Á.E., Hernández A. (2014). Prediction of Gas Permeability of Block-Segregated Polymeric Membranes by an Effective Medium Model. J. Membr. Sci..

[27] Ebadi-Dehaghani H., Nazempour M. (2012). Thermal Conductivity of Nanoparticles Filled Polymers. Smart Nanopart. Technol..

[28] Matteucci S., Yampolskii Y., Freeman B.D., Pinnau I., Yampolskii Y., Pinnau I., Freeman B. (2006). Transport of Gases and Vapors in Glassy and Rubbery Polymers. Materials Science of Membranes for Gas and Vapor Separation.

[29] Thornton A.W., Nairn K.M., Hill A.J., Hill J.M. (2009). New Relation between Diffusion and Free Volume: I. Predicting Gas Diffusion. J. Membr. Sci..

[30] Soto C., Torres-Cuevas E.S., Palacio L., Prádanos P., Freeman B.D., Lozano Á.E., Hernández A., Comesaña-Gándara B. (2022). Gas Permeability, Fractional Free Volume and Molecular Kinetic Diameters: The Effect of Thermal Rearrangement on Ortho-Hydroxy Polyamide Membranes Loaded with a Porous Polymer Network. Membranes.

[31] Soto C., Carmona J., Freeman B.D., Palacio L., González-Ortega A., Prádanos P., Lozano Á.E., Hernandez A. (2022). Free Volume and Permeability of Mixed Matrix Membranes Made from a Terbutil-M-Terphenyl Polyamide and a Porous Polymer Network. Polymers.

[32] Swager T.M. (2008). Iptycenes in the Design of High Performance. Acc. Chem. Res..

[33] Long T.M., Swager T.M. (2003). Molecular Design of Free Volume as a Route to Low- K Dielectric Materials. J. Am. Chem. Soc..

[34] Wiegand J.R., Smith Z.P., Liu Q., Patterson C.T., Freeman B.D., Guo R. (2014). Synthesis and Characterization of Triptycene-Based Polyimides with Tunable High Fractional Free Volume for Gas Separation Membranes. J. Mater. Chem. A.

[35] Robeson L.M. (1991). Correlation of Separation Factor versus Permeability for Polymeric Membranes. J. Membr. Sci..

[36] Robeson L.M. (2008). The Upper Bound Revisited. J. Membr. Sci..

[37] Aguilar-Lugo C., Suárez-García F., Hernández A., Miguel J.A., Lozano Á.E., De La Campa J.G., Álvarez C. (2019). New Materials for Gas Separation Applications: Mixed Matrix Membranes Made from Linear Polyimides and Porous Polymer Networks Having Lactam Groups. Ind. Eng. Chem. Res..

[38] Kononova S.V., Lebedeva G.K., Gubanova G.N., Kruchinina E.V., Vlasova E.N., Afanas N.V., Popova E.N., Volkov A.Y., Bykova E.N., Zakharova N. (2023). V Effect of Hydroxyl-Containing Fragments on the Structure and Properties of Membrane-Forming Polyamide-Imides. Membranes.

[39] Kwac L.K., Kim B., Chang J. (2025). Comparison of the Properties of Polyimides Derived from Various Dianhydride and Diamine Monomers. RSC Adv..

[40] Smith Z.P., Sanders D.F., Ribeiro C.P., Guo R., Freeman B.D., Paul D.R., McGrath J.E., Swinnea S. (2012). Gas Sorption and Characterization of Thermally Rearranged Polyimides Based on 3,3’-Dihydroxy-4,4’-Diamino-Biphenyl (HAB) and 2,2’-Bis-(3,4-Dicarboxyphenyl) Hexafluoropropane Dianhydride (6FDA). J. Memb. Sci..

