# Supplementary material for: Linking Intrinsic Filler Properties to Gas Separation Performance in Polyimide-Based Mixed-Matrix Membranes
Source: Polymers (Basel). 2026 Jul 1;18(13):1645. doi: 10.3390/polym18131645 (PMC13363900; doi:10.3390/polym18131645)
Supplement: Supplementary file 1 [file polymers-18-01645-s001.zip › polymers-4395486-supplementary.pdf]

polymers

Supporting Information

Linking Intrinsic Filler Properties to Gas Separation Performance in Polyimide-Based Mixed Matrix Membrane

Alba Torres [1,2], Cenit Soto [1,2], Javier Carmona [1,2], Raúl Muñoz [2], Laura Palacio [1,2], Pedro Prádanos [1,2], Alberto Tena [1,2] and Antonio Hernández [1,2,*]

[1] Surface and Porous Materials (SMAP), Associated Research Unit to CSIC, Universidad de Valladolid, Facultad de Ciencias, Paseo Belén 7, E-47011 Valladolid, Spain; alba.torres@uva.es (A.T.); marveliacenit.soto@uva.es (C.S.); fcojavier.carmona@uva.es (J.C.); laura.palacio@uva.es (L.P.); pradanos@termo.uva.es (P.P.); a.tena@uva.es (A.T.)
[2] Institute of Sustainable Processes (ISP), Universidad de Valladolid, Paseo Prado de la Magdalena 3-5, 47011 Valladolid, Spain; raul.munoz.torre@uva.es
* Correspondence: antonio.hernandez@uva.es

Academic Editor: Rui Zhao

Received: 8 June 2026
Revised: 28 June 2026
Accepted: 29 June 2026
Published: 1 July 2026

Copyright: © 2026 by the authors. Licensee MDPI, Basel, Switzerland. This article is an open access article distributed under the terms and conditions of the Creative Commons Attribution (CC BY) license.

1. Polymer characterization

1.1. 6FDA-based polyimides

The chemical structure of the 6FDA-based polyimides was confirmed by ^{1}H-NMR carried out in a Bruker Advance instrument working at 400 MHz and ATR-FTIR recorded by a PerkinElmer Spectrum One FT-IR (PerkinElmer, Waltham, MA, USA) with 4 cm^{-1} of resolution.

Pi-HABAc: ^{1}H NMR (400 MHz, DMSO-d_6) δ 8.20 (d, 2H), 7.98 (d, 2H), 7.84 (s, 2H), 7.81 – 7.75 (m, 4H), 7.66 (d, 2H), 2.14 (s, 6H). FTIR (film): imide ν(C=O) at 1777 and 1716 cm^{-1}, imide ν(C–N) at 1368 cm^{-1}.

Pi-DAPOH: ^{1}H NMR (400 MHz, DMSO-d_6) δ 10.33 (s, 1H, OH), 8.20 (dd, 2H), 7.97 (d, 2H), 7.81 (dd, 2H), 7.42 (s, 1H), 7.16 (d, 2H). FTIR (film): alcohol ν(−OH) at 3700-3000 cm^{-1}, imide ν(C=O) at 1785 and 1714 cm^{-1}, imide ν(C–N) at 1360 cm^{-1}.

Pi-DAROH: ^{1}H NMR (500 MHz, DMSO-d^6) δ 10.20 (s, 2H), 8.19 (d, 2H) ,7.99 (d, 2H), 7.84 (m, 2H), 7.24 (s, 1H), 6.67 (s, 1H). FTIR (film): alcohol ν(−OH) 3700−3000 cm^{-1}, imide ν(C=O) at 1785 and 1714 cm^{-1}, imide ν(C−N) at 1360 cm^{-1}.

Pi-DAM: ^{1}H NMR (400 MHz, DMSO-d_6) δ 8.21 (d, 2H), 7.99–7.92 (m, 4H), 7.35 (s, 1H), 2.17 (s, 4H), 1.95 (s, 2H). FTIR (film): imide ν(C=O) at 1788 and 1723 cm^{-1}, imide ν(C–N) at 1355 cm^{-1}.

PIM-1: ^{1}H NMR (400 MHz, DMSO-d_6): 6.8 (s, 2H), 6.5 (s, 2H), 2.1−2.4 (m, 4H), 1.2−1.4 (m, 12H). FTIR (film): ν(C−H) at 2900−2800 cm^{-1}, ν(−CN) at 2240, and ν(C−O) at 1262 cm^{-1}.

Number average molecular weight (M_n) and average molecular weight (M_w) respectively, were determined by gel permeation chromatography (GPC). Polyimide solutions in DMF + 0.1 % LiBr were formed and analyze by size exclusion chromatography using a Waters permeation chromatograph equipped with a Waters 2414 refractive index detector (Waters, Milford, MA, USA) and a set of Styragel HR3 and HR5 Waters columns. For calibration, polystyrene standards (Polymer Laboratories, Church Stretton, UK) were used. PIM-1 was dissolved and analyzed in THF using a set of HR4, HR1, and HR0.5 Waters columns with HPLC-grade.

Table S1. Number and Molecular weight and polydispersity for the synthesized polyimides.

Polymer	M_n (g mol^{-1})	M_w (g mol^{-1})	PI
Pi-HABAc	14862	24103	1.62
Pi-DAPOH	36288	89781	2.74
Pi-DAROH	13617	23788	1.75
Pi-DAM	17034	25298	1.49
PIM-1	18750	116040	6.19

1.2. Porous organic fillers

As an example, the synthesis for the Is-TPB POP is described below.

A 1.5/1 molar ratio of isatin and 1,3,5- triphenylbenzene were suspended in chloroform in a three-necked flask with a mechanical stirrer and nitrogen atmosphere. The mixture was stirred at room temperature under a nitrogen atmosphere, and after the mixture was cooled down to 0 °C, a 2:1 molar ratio TFSA was added dropwise. Then, the reaction was allowed to reach room temperature and stirred for 5 days. The solid formed was then poured into a water/ethanol mixture (3/1), filtered, and consecutively washed with water, acetone, and chloroform to eliminate unreactive chemicals. Finally, the product was milled and dried at 150 °C for 12 h under vacuum.

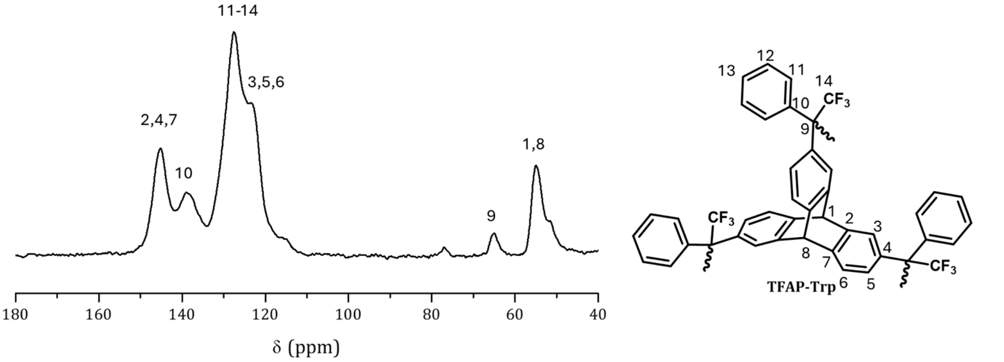

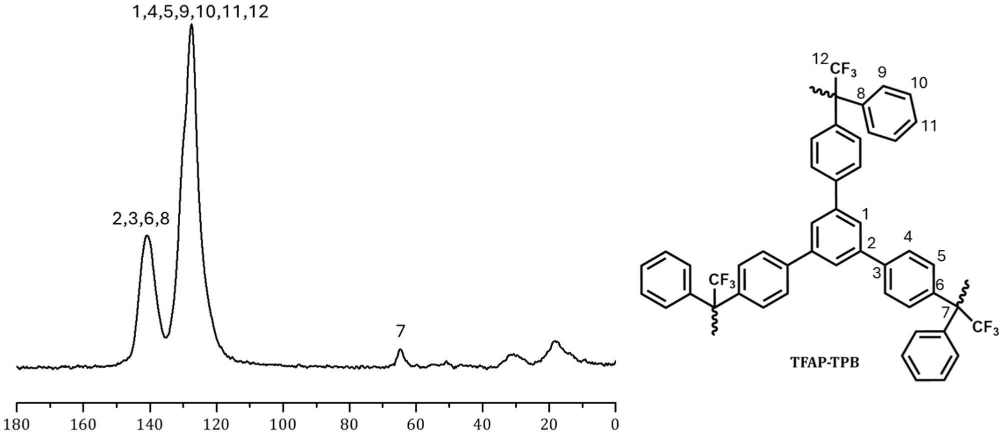

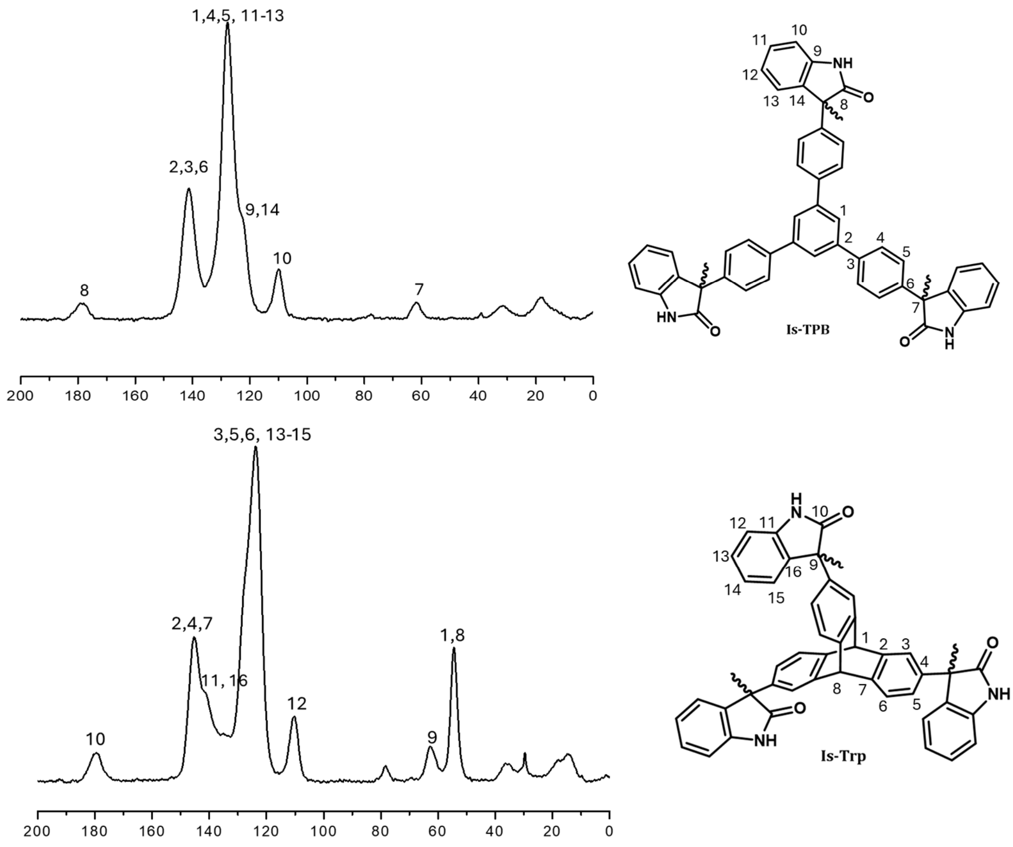

Figure S1. $^{13}C_{solid}$-RMN spectra for porous fillers.

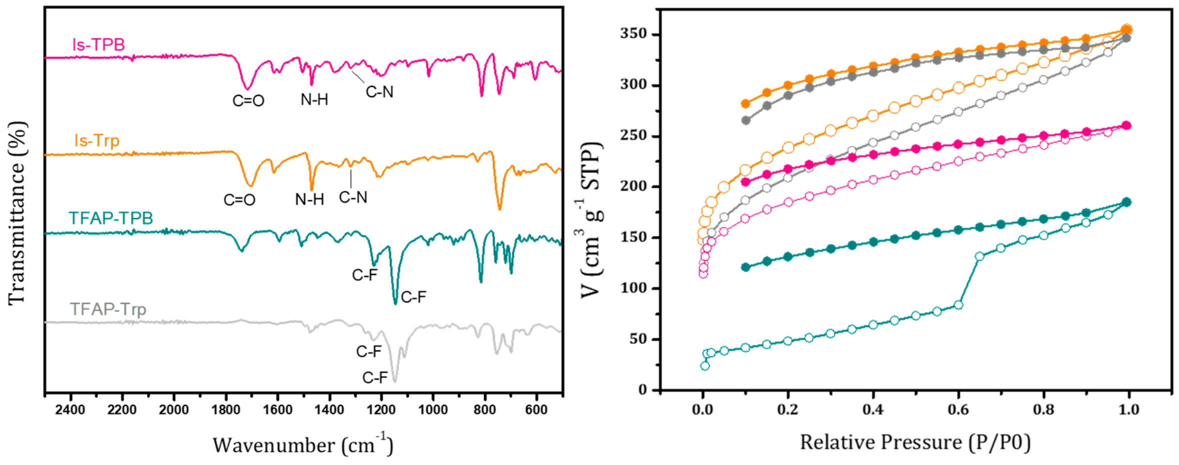

(**a**) (**b**)

Figure S2. (**a**) ATR-FTIR spectra for porous fillers. (**b**) N_2 isotherms at 77 K of the porous fillers

2. Membrane characterization

2.1. Attenuated total reflectance fourier transform infrared spectroscopy

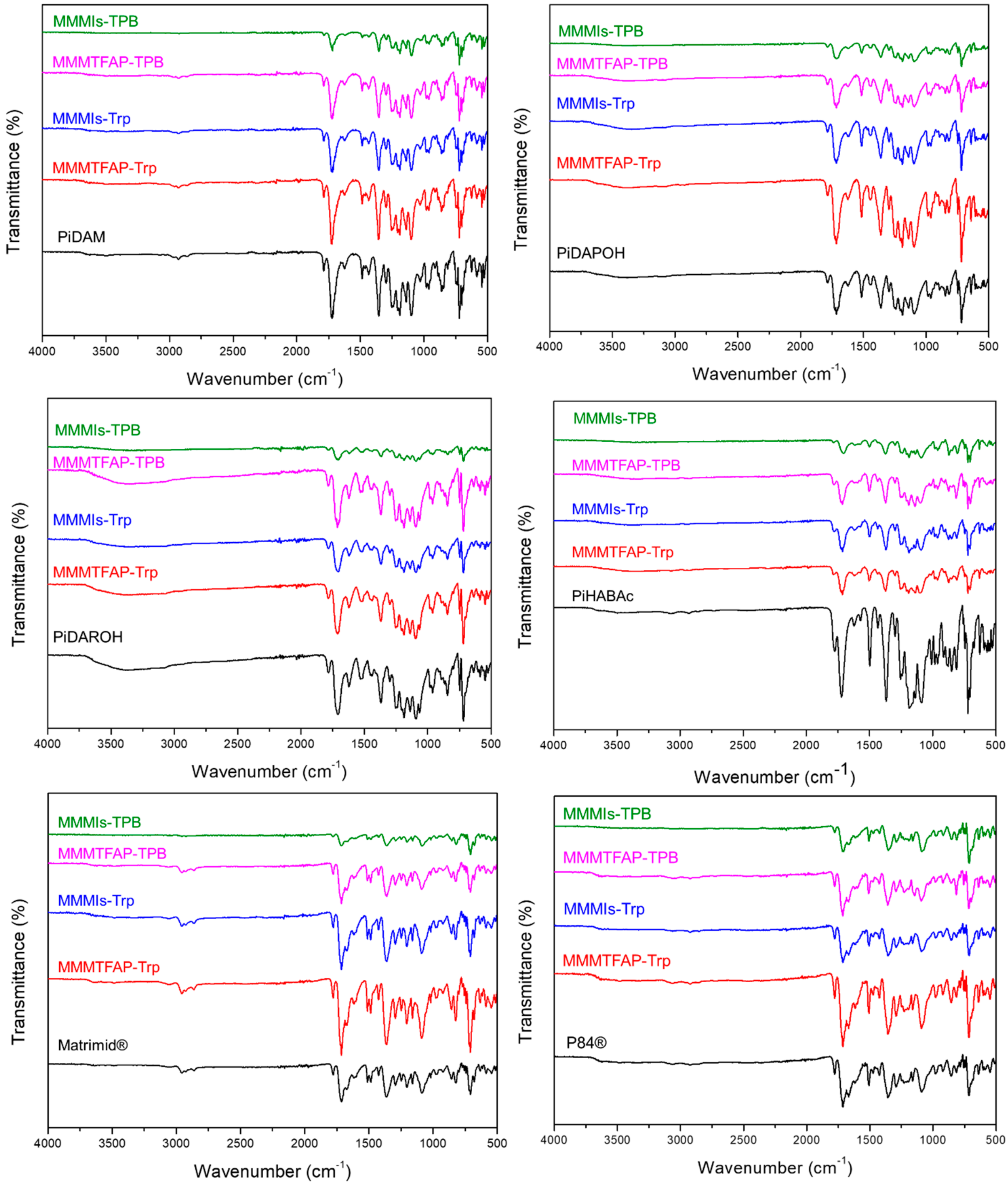

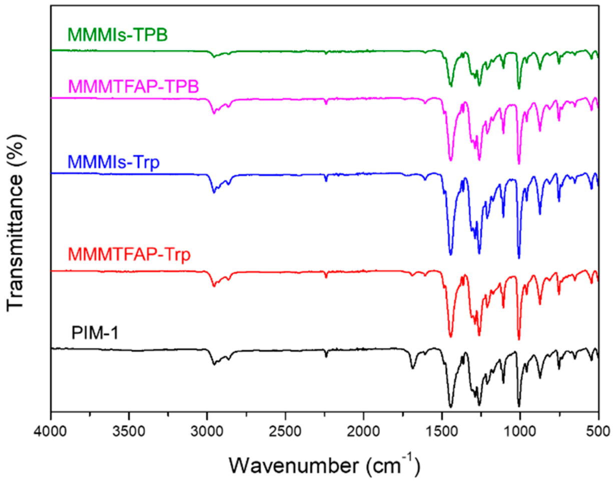

Figure S3. Comparative ATR-FTIR spectra between neat polymer and the respective MMMs.

2.2. *Thermogravimetric analysis*

Glass transition temperatures (T_g) and degradation temperatures (T_d) of all membranes are collected in Table S.I.2 and S.I.3. T_d were determined as the temperature where 5 % from the initial weight was recorded. As Pi-HABAc, Pi-DAPOH and Pi-DAROH can undertake a thermal rearranged (TR) process at temperatures between 275 and 450 °C, thermal rearrangement and the degradation of the polymer overlap. Therefore, T_d was determined as the onset point of the second weight loss.

Table S2. Glass transition temperatures (T_g) for all membranes.

	T_g (°C)				
**Matrix**	**Neat**	**TFAP-Trp**	**Is-Trp**	**TFAP-TPB**	**Is-TPB**
P84®	325	329	328	335	327
Matrimid®	324	328	328	329	328
Pi-HABAc	267[40]	328	320	260	341
Pi-DAPOH	355	371	360	371	334
Pi-DAROH	329	309	308	309	314
Pi-DAM	397	402	395	398	394
PIM-1	nd	nd	nd	nd	nd

Table S3. Degradation temperatures (T_d) for all membranes.

	T_d (°C)				
**Matrix**	**Neat**	**TFAP-Trp**	**Is-Trp**	**TFAP-TPB**	**Is-TPB**
-	-	491	521	495	563
P84®	505	508	503	504	499
Matrimid®	501	502	506	510	523
Pi-HABAc	513[a]	506[a]	514[a]	514[a]	517[a]
Pi-DAPOH	524[a]	522[a]	524[a]	515[a]	520[a]
Pi-DAROH	513[a]	511[a]	513[a]	500[a]	504[a]
Pi-DAM	506	508	503	511	509
PIM-1	505	501	501	500	502

[a]Calculated as the *Onset point* of the last weight loss step in TGA analysis.

https://doi.org/10.3390/polym18131645

2.3. Wide-angle X-ray scattering

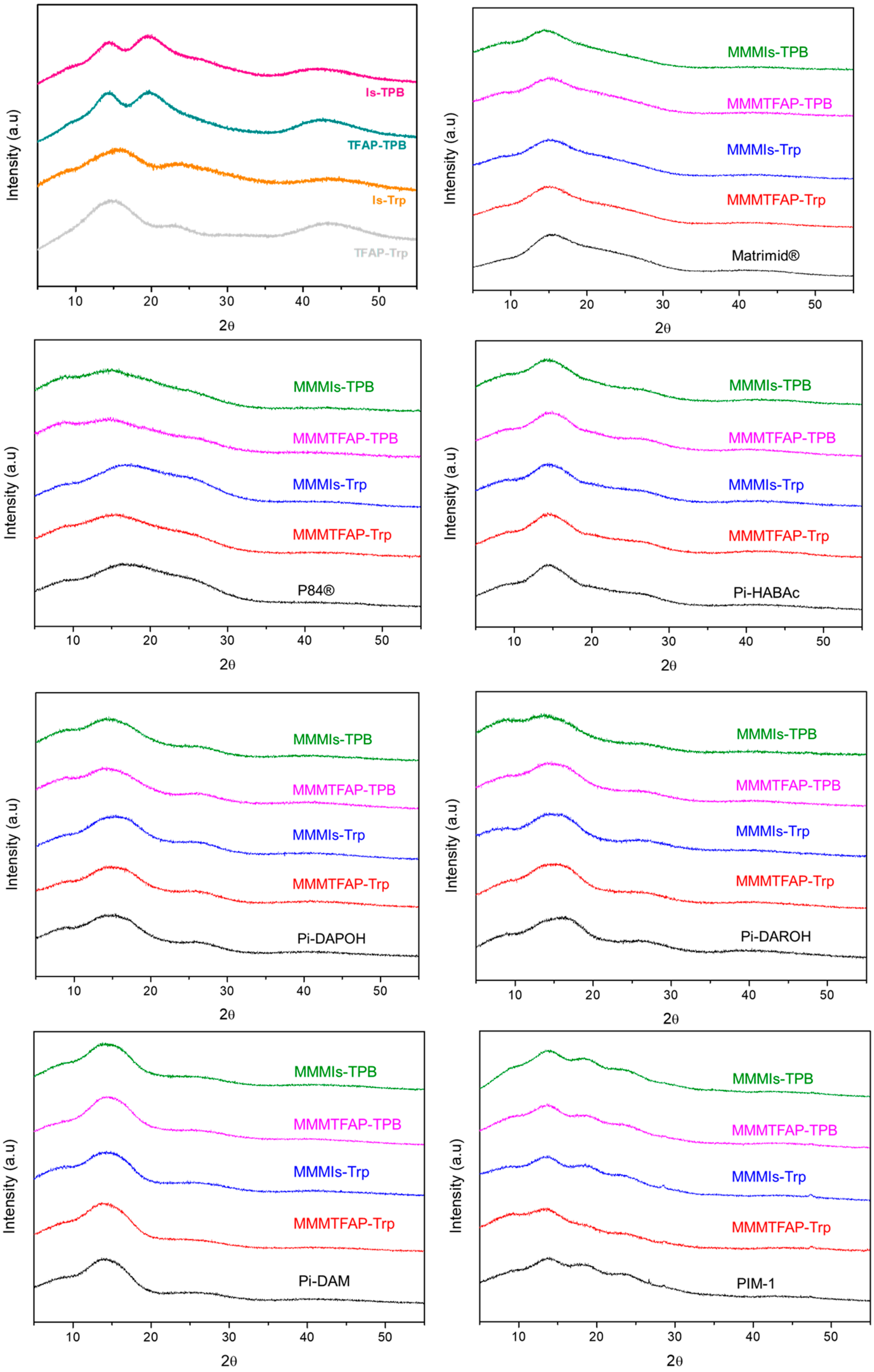

Figure S4. WAXS spectra for all membranes and fillers.

2.4. Permeability Coefficients

Table S4. Permeability coefficients (P) along with CO_2/CH_4 and O_2/N_2 selectivity measured at 3 bar and 35 °C for all neat and MMMs.

	P_{He}	P_{O2}	P_{CO2}	α (CO_2/CH_4)	α(O_2/N_2)
Matrimid®	21.5	1.81	7.98	32.2	6.1
TFAP-Trp–$MMM_{Matrimid®}$	33.1	2.95	13.5	33.8	6.1
Is-Trp–$MMM_{Matrimid®}$	21.9	2.37	11.4	30.8	5.6
TFAP-TPB–$MMM_{Matrimid®}$	23.5	2.47	12.1	31.9	5.9
Is-TPB–$MMM_{Matrimid®}$	34.4	3.30	16.4	44.3	6.3
P84®	9.51	0.29	1.04	53.0	9.7
TFAP-Trp–$MMM_{P84®}$	20.5	0.92	3.76	62.7	8.4
Is-Trp–$MMM_{P84®}$	14.5	0.49	1.95	55.8	9.5
TFAP-TPB–$MMM_{P84®}$	12.6	0.49	1.95	48.8	8.2
Is-TPB–$MMM_{P84®}$	15.8	0.84	3.8	54.3	9.3
Pi-HABAc	39	2.36	8.76	43.8	6.4
TFAP-Trp–$MMM_{Pi-HABAc}$	71.8	5.69	23.1	48.1	5.7
Is-Trp–$MMM_{Pi-HABAc}$	55.0	3.84	15.3	51.0	6.1
TFAP-TPB–$MMM_{Pi-HABAc}$	46.0	3.04	22.5	68	6.3
Is-TPB–$MMM_{Pi-HABAc}$	67.0	5.40	22.5	35.7	5.2
Pi-DAM	257	72	311	20.2	3.7
TFAP-Trp–MMM_{Pi-DAM}	304	94	446	21.4	3.7
Is-Trp–MMM_{Pi-DAM}	257	55.1	260	27.1	4.1
TFAP-TPB–MMM_{Pi-DAM}	214	58.6	265	22.0	3.8
Is -TPB–MMM_{Pi-DAM}	321	94.0	438	21.9	3.9
Pi-DAPOH	41.4	1.6	5.2	74	7.0
TFAP-Trp–$MMM_{Pi-DAPOH}$	61.6	3.12	11.5	77	6.9
Is-Trp–$MMM_{Pi-DAPOH}$	45.6	2.18	8.07	81	6.8
TFAP-TPB–$MMM_{Pi-DAPOH}$	48.4	2.35	7.99	80	7.8
Is -TPB–$MMM_{Pi-DAPOH}$	60.8	3.62	14.2	71	6.7
Pi-DAROH	42.0	1.8	6.86	76	6.7
TFAP-Trp–$MMM_{Pi-DAROH}$	72	3.58	14.2	101	6.8
Is-Trp–$MMM_{Pi-DAROH}$	61.4	3.47	12.9	129	8.3
TFAP-TPB–$MMM_{Pi-DAROH}$	68	3.59	13.7	98	7.0
Is -TPB–$MMM_{Pi-DAROH}$	88	5.60	22.3	72	6.4
PIM-1	1170	1123	5903	7.3	2.5
TFAP-Trp–MMM_{PIM-1}	1468	1560	8078	6.9	2.4
Is -Trp–MMM_{PIM-1}	1485	1449	7466	7.7	2.6
TFAP-TPB–MMM_{PIM-1}	1530	1462	6922	7.1	2.6
Is-TPB–MMM_{PIM-1}	1756	1804	9616	8.75	2.7

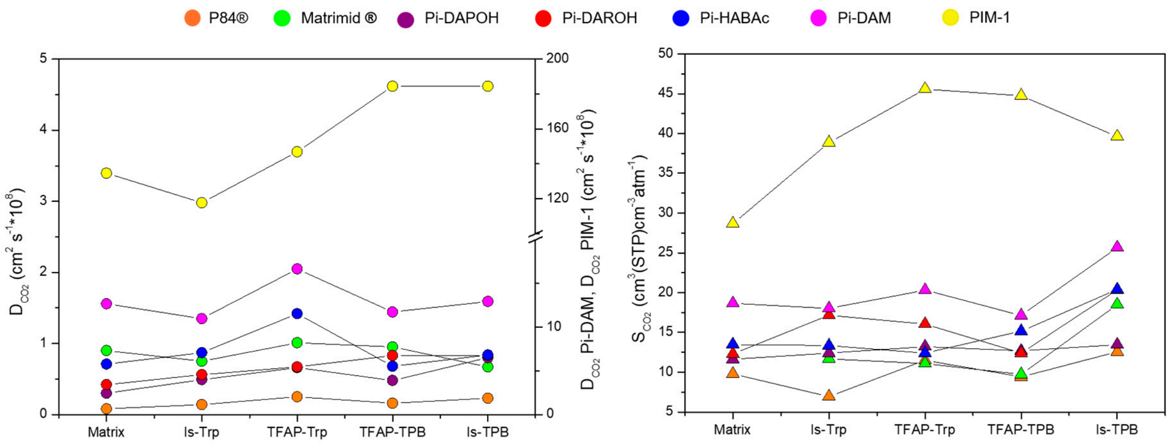

(**a**) (**b**)

Figure S5. (**a**) Diffusion, D, and (**b**) solubility, S, coefficient for CO_2 in function of particle inclusions.

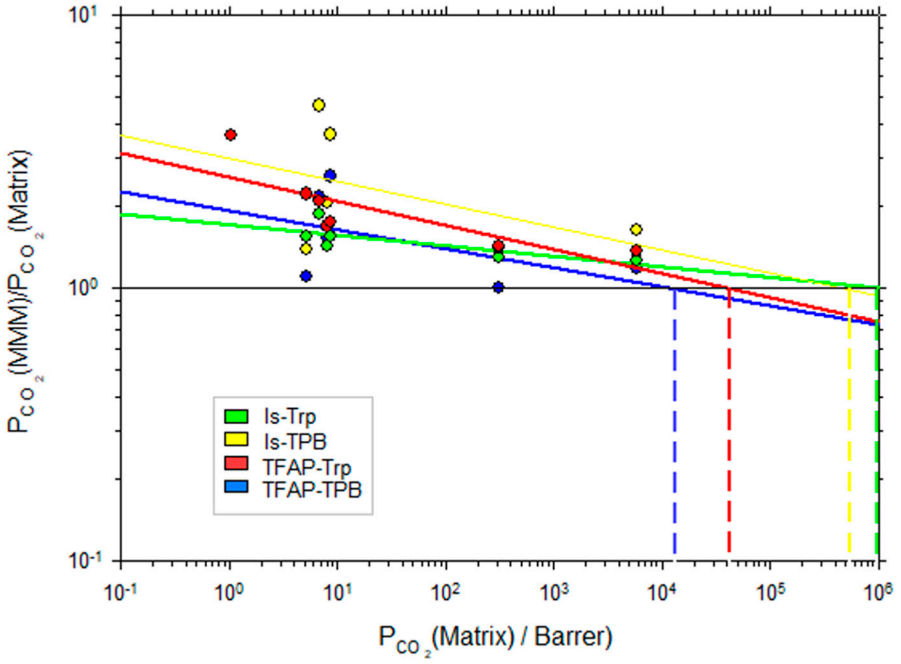

Figure S6. The ratio of the MMM CO_2 permeability over the corresponding pristine membrane as a function of permeability of the pristine (matrix) membrane. The fitted straights are shown for each filler.

Disclaimer/Publisher's Note: The statements, opinions and data contained in all publications are solely those of the individual author(s) and contributor(s) and not of MDPI and/or the editor(s). MDPI and/or the editor(s) disclaim responsibility for any injury to people or property resulting from any ideas, methods, instructions or products referred to in the content.

https://doi.org/10.3390/polym18131645
